# Dynamic neural networks: advantages and challenges

**DOI:** 10.1093/nsr/nwae088

**Published:** 2024-03-07

**Authors:** Gao Huang

**Affiliations:** Department of Automation, Tsinghua University, China

## Abstract

This perspective article delves into the transformative realm of dynamic neural networks, which is reshaping AI with adaptable structures and improved efficiency, bridging the gap between artificial and human intelligence.

Deep neural networks (DNNs) have underpinned the development of numerous contemporary artificial intelligence (AI) systems. Like the biological neural network, they comprise a substantial number of nodes and connections. The rapid advancement of computational hardware in the last decade has led to a significant expansion in the scale of DNNs, approaching the complexity of the human neural system. For instance, the GPT-3 model developed by OpenAI has achieved 175 billion parameters [1], and the human brain is estimated to consist of 86 billion neurons, interconnected by ∼100 trillion connections [2].

While recent large language models have exhibited remarkable performance, their efficiency lags behind that of the human neural system. For instance, the human brain consumes approximately 12 W [3]. In contrast, training a large language model (LLM) may require hundreds of machines operating for months, consuming millions of kilowatt hours of power. Performing inferences with LLMs is also computationally demanding, and the power consumption throughout their lifetime will be significant.

The question of why human brains are exceptionally efficient has sparked significant research interest in the field of AI. One apparent reason is that human brains operate with highly efficient bio-pulse signals, and only a small fraction of neurons would fire simultaneously. As a result, the information processing procedure is lightweight, despite the vast number of neurons. In contrast, DNNs function in a static manner: for any input, the model activates all its components when generating the output response.

Dynamic neural networks represent an emerging research focus within deep learning [4]. Different from conventional deep learning models with fixed computational graphs and parameters during inference, dynamic networks can adjust their structures and parameters in response to varying inputs, resulting in significant advantages regarding accuracy, computational efficiency, adaptability and more. Consequently, dynamic networks have garnered substantial research attention in recent years within the fields of computer vision, natural language processing and speech recognition.

## CATEGORIZATION OF DYNAMIC NEURAL NETWORKS

In general, dynamic neural networks can be categorized into three primary groups based on their methods for conducting conditional computations on input samples.

*Sample-wise* dynamic neural networks refer to deep learning models that dynamically allocate computation resources based on each individual input. Specifically, these networks treat each sample as a whole and do not delve into the internal data structure of individual samples. This characteristic distinguishes it from the other two types of dynamic models introduced below. Generally, sample-wise dynamic networks can be subdivided into two distinct categories.

The first category is implemented using a *dynamic architecture*, wherein the network comprises different segments to be executed conditioned on its input. The most common dynamic architecture is the early-exit network [5], which has multiple intermediate classifiers attached to various internal layers. The forward propagation can be halted when adequate confidence is achieved or when specific criteria are met at an internal classifier. Since the depth varies adaptively according to the input, we also refer to this model type as a dynamic depth network. Likewise, a network can exhibit dynamic width, typically implemented using a ‘gating’ mechanism, wherein a gating function is trained to regulate the activation of parallelly organized modules [6]. A more general variant is known as dynamic routing, where a router function is trained to determine the computation graph in a more adaptable manner [7]. Among the three aforementioned types of dynamic architectures, the dynamic depth network is the most commonly adopted, primarily owing to its simplicity in terms of training.

The second approach to implementing sample-wise dynamic networks involves incorporating dynamic parameters. One of the most well-known dynamic parameter networks is a model with an attention mechanism. Since attention weights are a function of the input, their values are dynamic, and we view a neural network with an attention mechanism as a dynamic model. It is also possible to directly predict certain sets of network weights to construct a dynamic parameter network [8]. A key finding regrading the introduction of dynamic parameters is its remarkable ability to enhance network capacity with minimal computational overhead.

In general, dynamic architectures are less compatible to batch computation, i.e. each sample has a distinct computation graph, making it challenging to efficiently process multiple samples in parallel. However, this may not pose a problem in specific scenarios, such as mobile computing, where inputs arrive sequentially, and there is no requirement for batch processing. In contrast, networks with dynamic parameters are more hardware-friendly. For instance, transformers with an attention mechanism can be efficiently deployed on contemporary GPUs.

*Spatial-wise* dynamic neural networks are models that consider the spatial structure within input samples, primarily applied to visual data like images [9] or point clouds [10]. Conventional deep learning algorithms distribute computation uniformly across spatial regions, resulting in redundant processing in many vision tasks, such as object detection, where the region of interest constitutes only a small fraction of the input. By adaptively concentrating on the informative regions that are most relevant to the task, spatial-wise dynamic networks can significantly enhance computational efficiency. Note that the practical efficiency of spatial-wise dynamic networks is sensitive to the granularity of spatially adaptive computation.

*Temporal-wise* dynamic neural networks can unevenly allocate computation along the temporal dimension for sequential data, such as videos [11] or time series data [12]. In the case of streaming data, such as videos, there is typically high correlation among nearby frames. Consequently, the dynamic focus on specific key frames is a crucial characteristic for deep learning models to reduce redundant computation. Furthermore, temporal-wise and spatial-wise adaptive computation could be implemented simultaneously to achieve higher efficiency.

## ADVANTAGES

Owing to their adaptive computing mechanisms, dynamic neural networks offer numerous notable advantages, as detailed below.

*Efficiency* is arguably the most prominent advantage of dynamic networks. In some cases, dynamic inference can improve the speed by more than one magnitude when achieving the same accuracy as a static model. There is a widespread belief that large neural networks involve a substantial number of redundant parameters and computational processes. This is the reason why model compression techniques such as neural pruning, weight quantization and model distillation have garnered substantial interest in both academia and industry. Notably, the dynamic neural network addresses the computational redundancy from quite a distinct perspective, and is compatible with the aforementioned methods.

*Adaptiveness* represents another favorable property of dynamic models, which is often absent in static models. Typically, the inference cost of a train neural network is constant for any input sample. Nevertheless, many applications require real-time adjustment of the trade-off between speed and accuracy. Many dynamic models, including early-exit networks, can fulfill this demand easily by dynamically adjusting some thresholds on the fly.

*Capacity.* Because of the adaptive computation paradigm, dynamic networks can exploit their parameters more thoroughly to learn more complicated representations compared to static models. In other words, dynamic models usually have a higher model capacity than those that do not employ a dynamic computation graph. Notably, the mixture-of-experts mechanism can expand the model parameters by eight times while maintaining similar computational cost, which significantly improves the performance of various vision tasks.

*Interpretability.* Spatial-wise and temporal-wise dynamic networks, by their ability to redirect attention to specific regions or time slots in the input, can provide insights into the information the model relies on when making decisions. This capability can be valuable in applications where decision transparency is crucial. Moreover, dynamic networks exhibit greater biological plausibility than static models, potentially opening up new avenues for exploring bio-inspired learning models and algorithms.

## CHALLENGES AND FUTURE DIRECTIONS

As a newly emerging research field, there are many open problems yet to be solved.

*Theory.* Dynamic computing introduces novel challenges that do not emerge in traditional machine learning theory. For example, theoretical formulation of the distribution shift problem among classifiers in an early-exit model remains unsatisfactory. In fact, the generalization properties, adversarial robustness and representation power of dynamic networks remain relatively underexplored in the literature.

*Optimization issues.* In the pursuit of adaptive computation, dynamic models typically incorporate discrete decision functions to be simultaneously learned alongside their continuous parameters, resulting in mixed-integer optimization challenges. Consequently, training dynamic networks requires specialized techniques, such as gradient approximation or the Gumbel-SoftMax trick. While the pre-train–fine-tuning paradigm and distillation technique have proven helpful in accelerating training and enhancing model performance, more efficient and effective approaches are sought to facilitate the training of a wide variety of dynamic networks.

*Hardware compatibility.* A major challenge in practical deployment of dynamic networks is the reduced degree of parallelism arising from the dynamic computation graph conditioned on each input or its constituent parts, leading to decreased efficiency on high-end GPU devices. Hence, it is imperative to design dynamic models that are more hardware-friendly from the algorithmic side, and it is equally valuable to develop hardware that is more compatible with dynamic computing. The combination with other hardware-friendly techniques, such as quantization and pruning, is also worth exploring. For example, the quantization precision could be adjusted conditioned on different inputs.

*Multi-modality modeling.* Recently, multi-modal foundation models, such as GPT-4 Vision, have demonstrated remarkable capabilities in processing natural language and visual input. Nevertheless, these models typically comprise billions, or even more, parameters, making them unaffordable or less economical for real-world applications. Hence, a valuable avenue for future research is developing efficient multi-modal foundation models based on dynamic networks.

In summary, there are still many research challenges that persist in the design, deployment and comprehension of dynamic networks. It is expected that these issues are likely to attract substantial research interest in the near future, given the remarkable advantages of dynamic models in terms of efficiency, efficacy and adaptiveness. Additionally, dynamic networks offer a promising solution to the low-power computation of large foundational models. Furthermore, the bio-inspired nature of dynamic networks has the potential to bridge the gap between deep learning models and the human brain. However, extensive research is still required to gain a deeper understanding and harness the full potential of dynamic networks in this context. It is anticipated that this will be a significant area of focus in the future.
